# Immunotherapy for Solid Tumors

**DOI:** 10.3390/cancers15061646

**Published:** 2023-03-08

**Authors:** Hortense de Saint Basile, Zineb Maaradji, Elizabeth Fabre

**Affiliations:** 1 APHP, Paris Cancer Institute CARPEM, Department of Thoracic Oncology, European Hospital Georges Pompidou, 75015 Paris, France; 2 INSERM Team UMR_S970, Immunotherapy and Antiangiogenic Treatment in Cancerology, Paris Descartes University, 75015 Paris, France

The development of immune checkpoint inhibitors (ICIs) constitutes a major therapeutic advance in the treatment of a number of malignancies. However, there remains a crucial need to explore the underlying mechanisms which impair the efficacy of immunotherapy.

This Special Issue includes four original articles and one review to investigate new strategies and predictors in order to rationalize the use of immunotherapy in the treatment of solid tumors.

Recently, the histone H3 lysine 36 (H3K36) methyltransferase NSD3, a neighbor gene of FGFR1, was identified as a key genetic driver of lung squamous cell carcinoma (LUSQ) [1]. By utilizing multi-scale analyses (genome, transcriptome, proteome, and TMA array), ref. [2] provides the first evidence that NSD3 gene amplification defines a non-immunogenic phenotype in LUSC and is associated with poor immunotherapy outcomes. Additionally, the authors demonstrate that high unfolded protein response (UPR) promotes the “cold” tumor immune microenvironment of NSD3-amplified LUSC. UPR signaling represents an intrinsic survival mechanism for tumor cells and has the capacity to potentially modify anti-tumor immunity [3].

By blocking immune checkpoints, ICIs disrupt immune homeostasis and can therefore potentially induce high immune reactivity and immune-related adverse events (irAEs). N. Guezour et al. [4] investigated the prognostic role of Grade 3–4 (irAE)s on overall survival in advanced NSCLC and showed that high-grade irAEs are clearly associated with better outcomes. The correlation between severe immune-related toxicity and outcomes in NSCLC brings important data for a better understanding of ICI mechanisms of actions.

Anti-programmed cell death protein-1 (PD1)/anti-programmed cell death protein-1 ligand (PDL1) have yielded disappointing results in a variety of situations, notably because of immune tolerance and low immunogenicity, as described in Ewing sarcoma (EwS) [5], an aggressive variety of bone tumor occurring in adolescents and young adults.

The development of challenging immune-based approaches, such as T-cell engagers, increases the interest in the identification of targetable tumoral surface antigenes. The article by K. Nagamura et al. [6] identifies G-protein-coupled receptor (GPCRs) GPR64 for the first time as a potential antibody-based therapeutic target for Ewing sarcoma. This research article offers perspectives for promising treatments in sarcomas through the identification of a targetable tumor antigens.

M. Kuske et al. [7] supported this Special Issue with a detailed review of the immunomodulatory effects of anti-PD1/PDL1 in immune cell types other than the T-cell compartment.

By describing the compensatory upregulation of non-targeted ICs, this article helps to understand resistance and irAEs mechanisms. Furthermore, the identification of interactions between different immune cell subsets provides the rationale for emerging ICI combinations.

Antacid agents such as proton pump inhibitors (PPIs) and histamine-2-receptor antagonists (H2RAs) are commonly prescribed owing to their potential capacity to modify the activity of anticancer therapies a variety of several mechanisms, including gut microbiome changes [8].

Rizzo et al. [9] performed a systematic literature review and meta-analysis to investigate the outcome of NSCLC patients receiving concomitantly antiacid agents and immunotherapy. The authors present pooled results of six studies into NSCLC patients treated by ICI, and report a reduced OS and PFS in the cases of PPIs or H2RAs use. The authors conclude that special consideration should be given to antiacid agents prescription in order to preserve the anti-tumor immunity through the promotion of microbiote integrity.

In conclusion, this Special Issue reports recent, highly relevant data to discuss how to predict and enhance responses to immunotherapy and thereby define new therapeutic strategies.
